# Influence of Non-Metallic Inclusions on Bending Fatigue Strength of High-Quality Carbon Constructional Steel Heated in an Industrial Electric Arc Furnace

**DOI:** 10.3390/ma15176140

**Published:** 2022-09-04

**Authors:** Tomasz Lipiński

**Affiliations:** Department of Material and Machine Technology, The Faculty of Technical Sciences, University of Warmia and Mazury in Olsztyn, 10-719 Olsztyn, Poland; tomaszlipinski.tl@gmail.com

**Keywords:** constructional steel, fatigue strength, non-metallic inclusions

## Abstract

Non-metallic inclusions are one of the many factors influencing the strength of materials operating under variable loads. Their influence on the strength of the material depends not only on the morphology of the impurities themselves, but it is also closely related to the microstructure of the material. This microstructure is the matrix for non-metallic inclusions. This article discusses the results of a study investigating the effect of non-metallic inclusions on the fatigue strength of structural steel during rotary bending. The study was performed at 12 heats produced in an industrial plant’s 140-ton electric furnaces. Six heats were desulphurised, and six were refined with argon. This paper presents the bending fatigue strength of steel hardened and tempered at different temperatures, subject to the relative volume of inclusions. This paper also presents the dimensional structure of non-metallic inclusions divided by different two technologies. The research shows that the main fraction of non-metallic inclusions is Al_2_O_3_; the most numerous were impurities with a diameter of less than 2 µm; argon refining does not affect the proportion of non-metallic inclusions of large dimensions (with a diameter of over 15 µm); the influence of non-metallic inclusions on the strength of the steel is also related to the microstructure of the steel constituting the matrix of inclusions.

## 1. Introduction

For the machines and devices produced today, the user requires a low failure rate and the minimisation of maintenance costs in the operation process. These requirements translate into the quest to provide high-quality construction materials [1,2,3,4].

The longevity and reliability of machines mainly depend on the quality of the construction materials, the environment and working conditions, and the construction solutions of the facility. Service wear is a normal phenomenon that always accompanies materials in an operation process. It cannot be eliminated, but its course can be determined quite precisely, and it is highly predictable.

Unfortunately, failures and damage to machine parts are observed during operation. These events are unpredictable and random in nature. There are various reasons for the sudden failure of machine parts. These include material defects, construction imperfections, and human factors, as well as many other reasons [5,6,7,8,9,10].

Unfortunately, each increase in the quality of materials is accompanied by an increase in the cost of production [11]. It does not seem advisable to produce a material of the highest quality with a large margin of durability (exceeding the service life) without taking into account the costs of production. Nowadays, economic factors are becoming increasingly important. At the current level of technology and with the greater number of possibilities for shaping the quality of materials, there is a tendency to produce materials with a certain period of failure-free operation accompanied by the greatest possible reduction of production costs [5,11,12,13,14].

Analyses of the causes of the premature failure of machine parts often indicate that material fatigue was the cause [15,16,17]. The properties of the material are due to its microstructure. Therefore, the possibilities of using the material depend on the microstructure [18,19,20,21]. Steel has been the most popular construction material for decades. The properties of high-quality steels are mainly due to the chemical composition and production process [22,23,24,25,26,27,28]. In addition to its main chemical components, steel also contains impurities. Despite their small quantities, they play an important role, especially in high-quality steels. Much importance is also attached to inclusions. The importance of both impurities and inclusions in steel depends on their type, shape, quantity, distribution, etc. [29,30,31] Many authors report that non-metallic inclusions in high-grade steel adversely affect fatigue strength. Their impact depends on the size and shape, the density of occurrence, and their location in relation to the grain boundaries of the material, etc. [32,33,34,35,36] Some authors also report that minor non-metallic inclusions need not reduce the fatigue strength of the steel [29].

The resistance to variable loads results not only from the stress level, load cycle, and amplitude, but also from the microstructure of the steel and the participation of non-metallic phases [37,38,39,40]. The steelmaking practice affects the relative volume and quality of non-metallic inclusions. In order to reduce the content of impurities, the outside furnace treatment of steel is also carried out [23,24,25]. Although the relative volume of non-metallic inclusions in high-purity steel is very small, these impurities can have a significant effect on the properties of the alloy produced [39,41,42,43]. Many different technologies have been developed to minimize the proportion, by volume and quantity, of contaminants in order to improve the fatigue properties of steel. Despite the high technological level, no method has yet been developed that would allow the industry to cheaply and effectively remove non-metallic inclusions [38,44,45]. Mainly on the basis of laboratory scale tests, it has been confirmed that the fatigue strength of steel depends mainly on the volume, quality, size, shape, and distribution of the impurities. The authors of such studies usually conduct tests on steels of a high hardness. Studies of steels with high ductility are not usually considered to be mainstream research [35,42,46]. Contamination in high-purity steels, due to its limited volume and dimensions, affects the fatigue life of the material through interactions in micro-areas, which are closely related to the microstructure as a matrix of inclusions [29,30,47]. 

Despite the development of analytic techniques and computer systems [48], it is not easy to relate these parameters, and, due to this, the research topic will continue to remain important.

The main aim of this research was to determine the influence of the relative quantity of impurities on the fatigue strength of carbon steels of different levels of hardness and plasticity.

## 2. Materials and Methods

The average chemical composition of the analysed steel is presented in Table 1.

The experimental material consisted of high-quality carbon constructional steel heated in an industrial electric arc furnace using two metallurgical technologies. Due to the use of various technologies, the melts had different relative volumes and sizes of non-metallic inclusions. In order to obtain the different properties of the steel, heat treatment was carried out, as a result of which, different microstructures were obtained. 

In the E technology, steel was melted in an arc furnace with a capacity of 140 tons. Six industrial melts were carried out, and the desulphurisation process was conducted. The molten metal was poured into a 7-ton steel ladle. In the classic fashion, caissons with a cross-section of 100 mm × 100 mm were rolled from the ingots.

In the EA technology, the steel was melted, as in the E technology, and it was then poured into the ladle and refined with argon. Argon was introduced into the liquid steel through a porous brick. The argon purging time ranged from 8 to 10 min. After the refining process, profiles were produced, as in the E technology.

The chemical composition was determined for each of the heats using a LECO quantometer and traditional chemical analysis methods. The relative volume of non-metallic inclusions with a minimum diameter of 2 µm was determined with a Quantimet video inspection microscope under 400× magnification. The relative total volume of non-metallic inclusions was determined by the chemical extraction method. The relative volume of inclusions in the range of up to 2 µm was calculated analytically by subtracting from the total volume of inclusions the volume obtained by image analysis with a diameter greater than 2 µm. The number of particles in the range of 2 μm and smaller was the difference between the number of all inclusions determined by chemical extraction and the number of inclusions measured by the video method. 

Calculations of the relative volume of non-metallic inclusions were carried out assuming that the quotient of particle surfaces and the observation area are equal to the quotient of the volume of particles in the assumed volume and the assumed volume.

For the fatigue strength tests, 51 sections with a cylinder cross-section of 10 mm in diameter were taken from 100 mm × 100 mm billets for each melt. The main axes of the rolls were parallel to the billet-rolling direction. The heat treatment, aimed at the production of various steel microstructures, consisted in austenitizing the samples at the temperature of 880 °C for 30 min. The samples were then chilled in water. The tempering process was carried out at temperatures of 200, 300, 400, 500, and 600 °C, annealing the samples for 120 min. After annealing, the samples were cooled in air. As a result of the applied heat treatment, the steel gained different microstructures, which resulted in different hardness, ranging from 271 HV to 457 HV, depending on the tempering temperature [28,46].

The fatigue-strength test was carried out on all samples from two metallurgical technologies. The test was carried out on a rotary bending machine with a frequency of 6000 revolutions per minute. A reference level of 10^7^ cycles was adopted to determine the fatigue strength. The loads of the individual samples depended on the strength of the steel. The fatigue-strength test was carried out by changing the load every 40 MPa. The maximum load values were selected experimentally. When selecting them, it was assumed that the fatigue limit would be reached at the level of 10^4^ to 10^6^ cycles. The following maximum loads were assumed for individual tempering temperatures: 650 MPa for 200 °C, 600 MPa for the range from 300 °C to 500 °C, and 540 MPa for 600 °C.

The fatigue strength for an individual tempering temperature is presented in the form of linear regression Equation (1):

*z_go_* (temperature tempered) = a · V + b, (1)

where:

*z_go_*—rotating bending fatigue strength, MPa,

V—relative volume of non-metallic inclusions, vol.%,

a, b—coefficients of the equation.

The significance of correlation coefficients r was determined on the basis of the critical value of the Student’s *t*-distribution for a significance level of α = 0.05, and the number of degrees of freedom f = *n* − 1 by Formula (2):

(2) $$t=\frac{r}{\sqrt{\frac{1-r^{2}}{n-1}}},$$

The diffusion coefficient *z_go_* for the regression equation was calculated using Equation (3):

(3) $$\delta =2s_{zgo}\sqrt{1-r^{2}},$$

where:

*s_zgo_*—standard deviation,

*r*—correlation coefficient.

## 3. Results and Discussion

**Figure 1:**
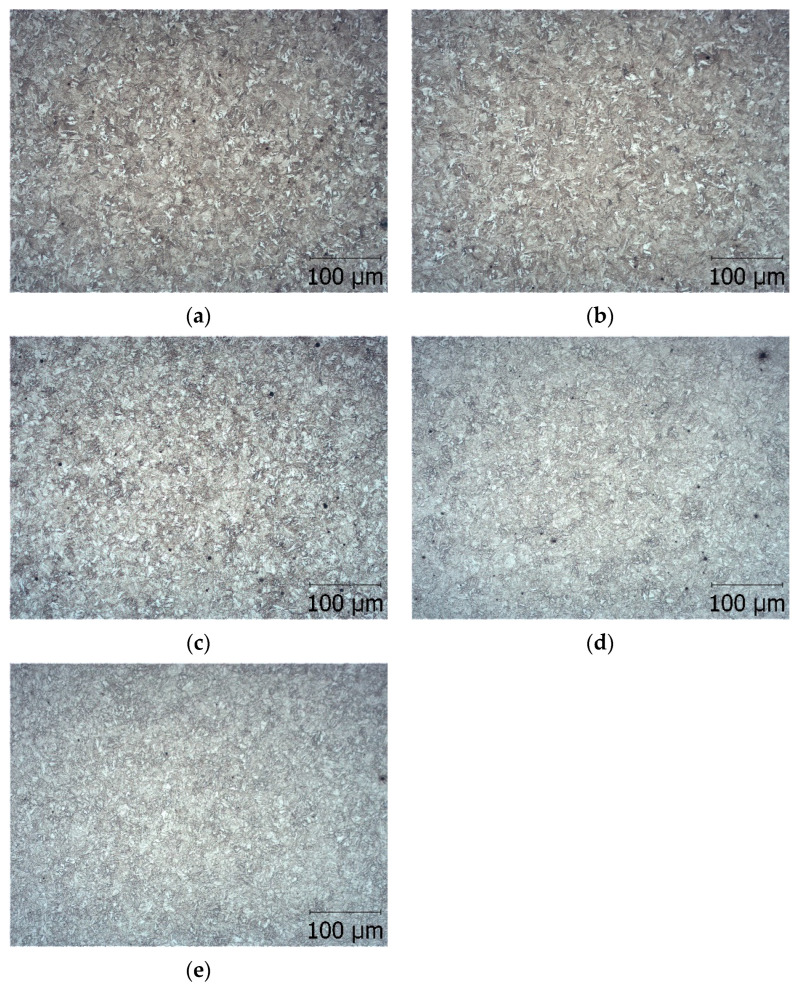
Microstructure of steel heated in an electric furnace and desulphurised (E technology), hardened at 880 °C, and tempered at: (**a**) 200 °C tempered martensite, (**b**) 300 °C tempered martensite with metastable carbides, (**c**) 400 °C tempered martensite with cementite formations, (**d**) 500 °C sorbitol, and (**e**) 600 °C spheroidite [49].

The microstructures of the tested steel after different heat treatments are presented in Figure 1.

We obtained microstructures with the low plasticity of low-tempered martensite, medium hardness and the plasticity of sorbitol, and the low hardness and high plasticity of spheroidite.

The statistical parameters representing the phase structure of the non-metallic inclusions are presented in Table 2. 

**Figure 2:**
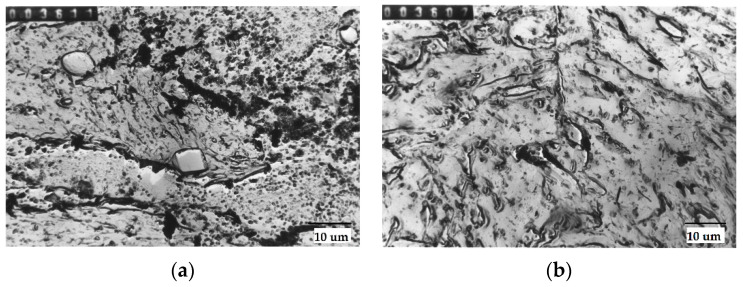
Non-metallic inclusion in tested steel: (**a**) spherical MgO and parallelepiped Cr_2_O_3_ heats produced in EA technology, (**b**) small spheroidal MgO [49].

Sample particles for tested steel are presented in Figure 2.

**Figure 3:**
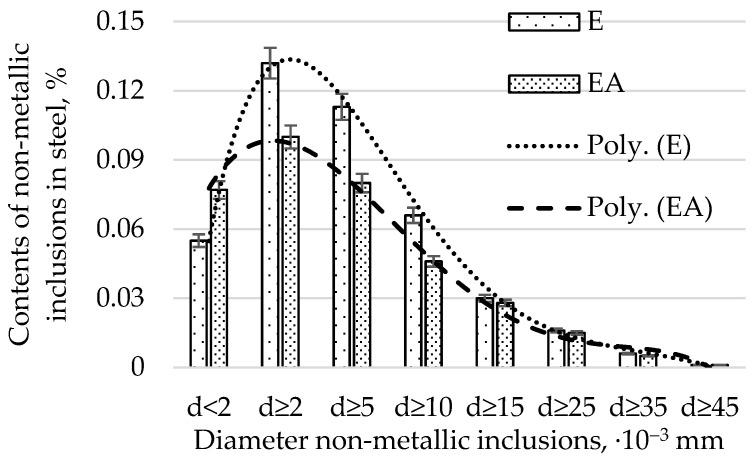
Average diameters of non-metallic inclusions from heats produced in electric furnaces using E and EA technology.

The average diameters of non-metallic inclusions from heats produced in electric furnaces using E and EA technology are presented in Figure 3.

**Figure 4:**
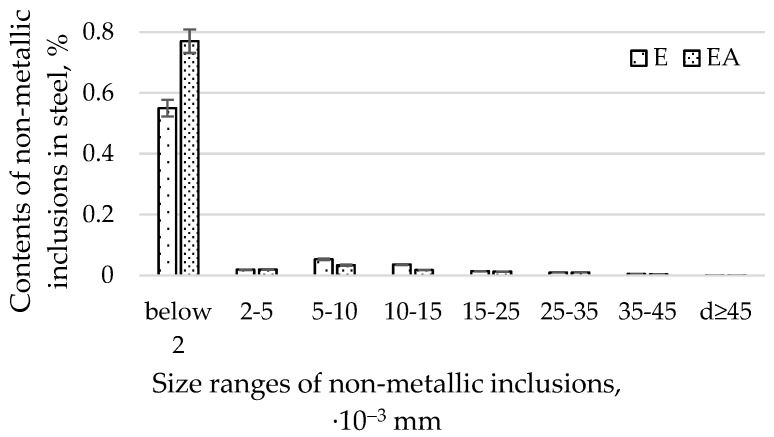
Average diameters of non-metallic inclusions from heats produced in electric furnaces using E and EA technology in size ranges for all sizes of impurities.

The average diameters of the non-metallic inclusions from heats produced in electric furnaces using E and EA technology in size ranges for all sizes of impurities are presented in Figure 4.

By analysing the relative volume of impurities with diameters of d ≥ 2, d ≥ 5, d ≥ 10, d ≥ 25, and d ≥ 35 µm (Figure 4), we found that the EA technology, in comparison with the E technology, produced a lower content of impurities. The difference in the contents of the non-metallic inclusions produced by the EA and A technologies decreases with an increase in the diameter of the impurities. Moreover, it was found that the highest relative volume of impurities (amounting to 0.132% for the E technology and 0.008% for the EA technology, respectively) occurs for impurities with a diameter of d ≥2 µm. One should note that the range above 2 µm is very wide. This fact results in the largest volume of impurities found in steel. For d ≥ 45 µm, the relative volume of impurities for both technologies was comparable. The distribution of contents of non-metallic inclusions for diameter ranges greater than 2 µm for both technologies resembles a normal distribution. The relative volume of non-metallic inclusions with diameters below 2 µm was smaller for technology E. It follows that the steel produced in the EA technology had more submicroscopic impurities (below 2 µm) and a comparable relative volume of large-diameter impurities (those with a diameter greater than 25 µm) compared to EA.

**Figure 5:**
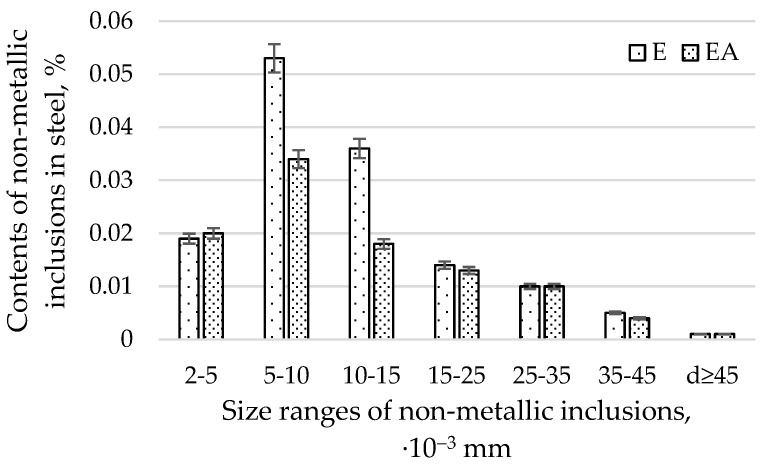
Average diameters of non-metallic inclusions from heats produced in electric furnaces using the E and EA technologies, in size ranges covering all sizes of impurities greater than 2 µm.

When analysing the relative volume of impurities in individual size ranges (Figure 4), a much greater proportion of impurities in the range with a diameter below 2 µm was found than in any of the remaining ranges representing individual ranges of diameters. For technology E, the content of non-metallic inclusions was 0.055%, and for EA, it was 0.077%. As mentioned previously, the volume of non-metallic inclusions for the remaining compartments, due to large differences in the diameters of non-metallic inclusions in relation to those with diameters below 2 µm, is definitely smaller. In order to improve readability, it was decided to present the ranges for diameters above 2 µm in Figure 5. An analysis of Figure 4 and Figure 5 shows that significant differences in the proportion of non-metallic inclusions for both technologies occurred for the following dimensional ranges: below 2 µm, from 5 to 10 µm, and from 10 to 15 µm. For the remaining ranges, the differences in the shares of pollutants were insignificant.

Figure 5 presents the average diameters of the non-metallic inclusions from heats produced in electric furnaces using the E and EA technologies, in size ranges covering all sizes of impurities greater than 2 µm.

**Figure 6:**
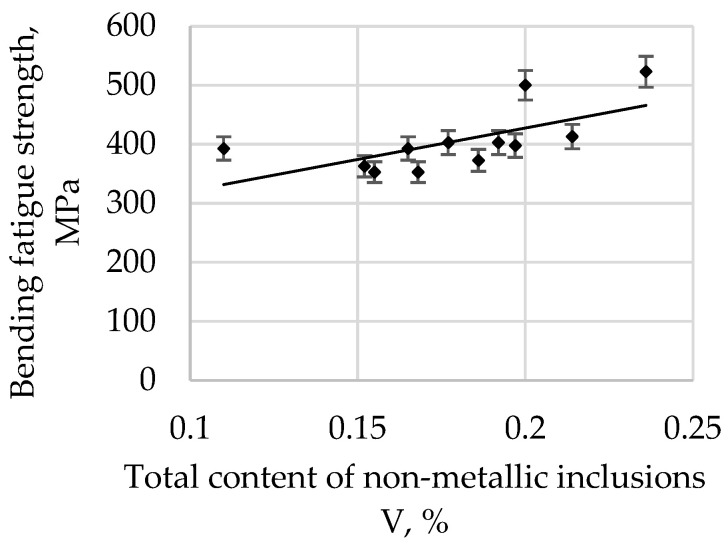
The bending fatigue strength of tested steel hardened and tempered at 200 °C depends on the total content of non-metallic inclusions.

The bending fatigue strength of high-quality carbon construction steel hardened and tempered at 200 °C depends on the relative total content of non-metallic inclusions, and this is presented in Figure 6.

**Figure 7:**
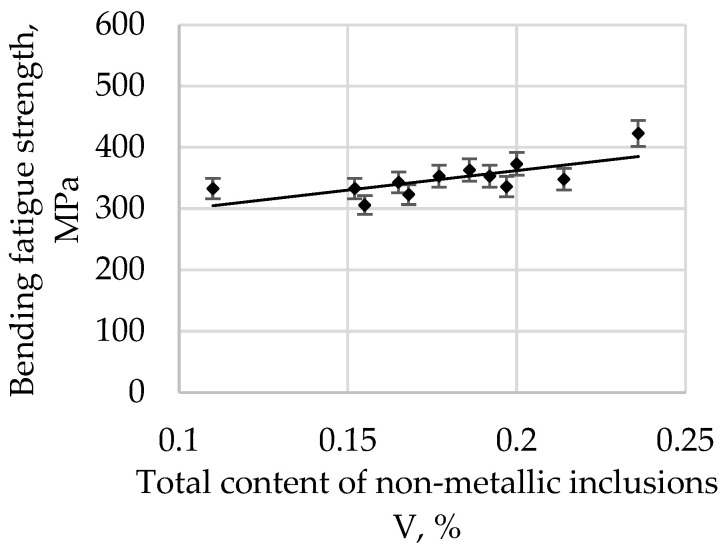
The bending fatigue strength of tested steel hardened and tempered at 300 °C depends on the total content of non-metallic inclusions.

The bending fatigue strength of high-quality carbon construction steel hardened and tempered at 300 °C in depends on the relative total content of non-metallic inclusions, and this is presented in Figure 7.

**Figure 8:**
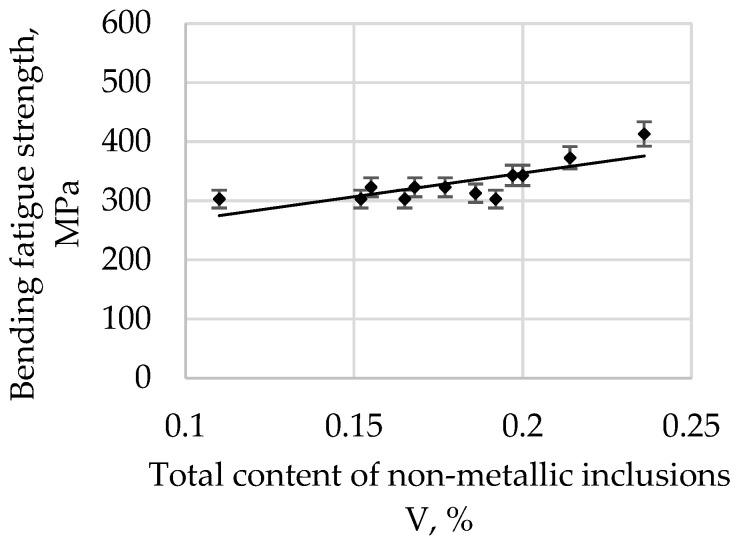
The bending fatigue strength of tested steel hardened and tempered at 400 °C depends on the total content of non-metallic inclusions.

The bending fatigue strength of high-quality carbon construction steel hardened and tempered at 400 °C depends on the relative total content of non-metallic inclusions, and this is presented Figure 8. 

**Figure 9:**
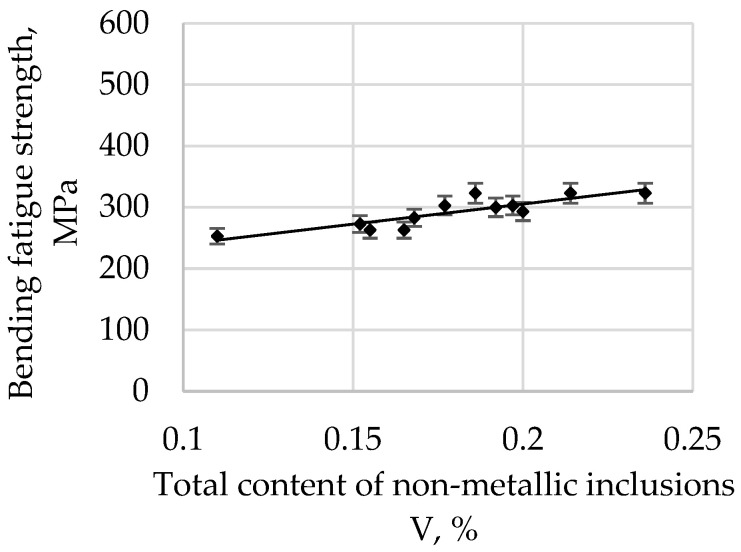
The bending fatigue strength of tested steel hardened and tempered at 500 °C depends on the total content of non-metallic inclusions.

The bending fatigue strength of high-quality carbon construction steel hardened and tempered at 500 °C depends on the relative total content of non-metallic inclusions, and this is presented Figure 9. 

**Figure 10:**
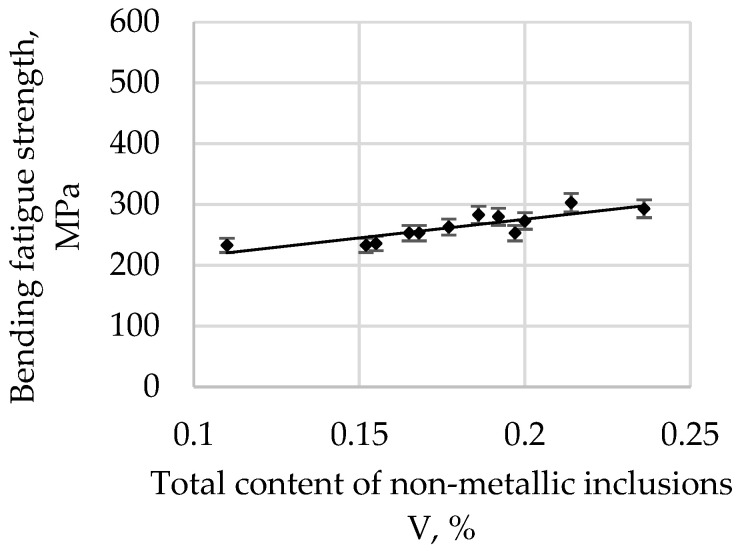
The bending fatigue strength of tested steel hardened and tempered at 600 °C depends on the total content of non-metallic inclusions.

The bending fatigue strength of high-quality carbon construction steel hardened and tempered at 600 °C depends on the relative total content of non-metallic inclusions, and this is presented Figure 10.

Parameters representing the relevant mathematical models and correlation coefficients are presented in Table 3.

The correlation coefficient r is 0.6506 for a tempering temperature of 200 °C, 0.7094 for 300 °C, 0.7831 for 400 °C, 0.8642 for 500 °C, and 0.8552 for 600 °C. As the tempering temperature increases, its value also increases. Therefore, it is possible to describe with sufficient statistical accuracy the linear function Equation (1) in Table 3.

All parameters representing the regression Equation (1) presented in Table 3 are statistically significant at the level of α = 0.05. The regression coefficient b (1) for the tempering temperature of 200 °C is 215.22, and it systematically decreases with an increase in the tempering temperature, reaching 152.77 for 600 °C. Based on these changes, it can be concluded that, with an increase in the tempering temperature, the bending fatigue strength decreases. It is known that, after low-tempering at 200 °C, the microstructure is predominantly thin-tempered martensite. As the tempering temperature increases, the martensite microstructure becomes, in turn, a medium and then a highly tempered martensite microstructure. It is also known that an increase in the tempering temperature results in a decrease in hardness due to changes in the martensite. Thus, the relations at the boundaries between non-metallic inclusions and matrices of different hardnesses and plasticities also change. The plastic matrix is able to absorb some of the energy generated at the matrix–pollution interface due to plastic deformation. One should also take into account the consideration that the tested steel belongs to the group of carbon steels, so the results should not be compared, for example, with hard bearing steels, for which the reaction at the inclusion–matrix interface may proceed in a different way.

An analysis of the bending fatigue strength of tested steel hardened and tempered at different temperatures depending on the total content of non-metallic inclusions shows that fatigue strength increases with an increasing relative volume of inclusions. It should be remembered, however, that the tested steel is of high purity and carbon content. As shown earlier in Figure 4, the main fraction of non-metallic inclusions includes impurities with diameters of less than 2 µm. At the same time, with such small diameters, if the relative volume of this fraction is high, it should be concluded that their quantity must also be large. (This fraction is by far the majority fraction found in steel.) When analysing the increase in strength as a function of the increase in volume of non-metallic inclusions, it should be assumed that submicroscopic inclusions are capable of slowing down the formation and development of microcracks. However, their interaction with the type of their matrix, i.e., the steel microstructure, should be inseparably linked. The presented study also confirms the results presented in [29].

## 4. Conclusions

The paper describes the influence of the relative quantity of impurities on the fatigue strength of carbon steel of different hardnesses and plasticities. 

Steel that has been heated in industrial conditions in an electric furnace, desulphurised, and refined with argon has a greater proportion of a relative volume of non-metallic inclusions with diameters below 2 µm. This steel also has a smaller proportion of non-metallic inclusions in the dimensional ranges of 5–10 µm and 10–15 µm.

No argon-refining changed the quantity of non-metallic inclusions with diameters greater than 15 µm in the steel.

In the tested, high-purity carbon steel, submicroscopic, non-metallic inclusions dominated in number but not in volume.

Submicroscopic non-metallic inclusions (with diameters below 2 µm) do not reduce the fatigue strength of high-purity carbon steel. It can even be assumed that they can lead to the later fatigue failure of the steel.

## Figures and Tables

**Table 1:** Real chemical composition of tested alloys [wt.%].

	C	Si	Mn	P	S	Cr	Ni	Mo	Cu	B
E	0.26	0.25	1.19	0.02	0.01	0.53	0.49	0.25	0.14	0.003
EA	0.23	0.29	1.22	0.02	0.01	0.47	0.46	0.23	0.15	0.003

**Table 2:** Phase structure of non-metallic inclusions [vol.%].

Technological Process	Statistical Parameter	Al_2_O_3_	SiO_2_	MnO	MgO	CaO	FeO	Cr_2_O_3_
E	arithmetic average	41.7	14.4	7.10	7.9	9.3	8.7	10.5
	standard deviation	2.01	1.68	1.95	1.75	1.27	2.97	2.08
EA	arithmetic average	39.3	13.2	8.5	9.7	10.3	9.6	9.1
	standard deviation	2.06	1.45	2.26	0.82	1.57	2.25	1.25

**Table 3:** Parameters representing relevant mathematical models and correlation coefficients.

Tempering Temperature °C	Regression Coefficient a (1)	Regression Coefficient b (1)	Correlation Coefficient r	Degree of Dissipation *z_go_* around Regression δ [MPa] (3)	t_α=0.05_ Calculated by (2)	t_α=0.05_ from Student’s *t*-Distribution for *p* = (*n* − 1)
200	1062	215.22	0.6506	81.33394572	2.841371571	
300	636.12	234.84	0.7094	41.47079098	3.338242423	
400	802.18	186.64	0.7831	41.80383589	4.176340892	2.201
500	656.55	174.17	0.8642	25.08698158	5.696583502	
600	614.64	152.77	0.8552	24.44294019	5.472482558	
